# Public Health Advocacy in Times of Pandemic: An Analysis of the Medicare-For-All Debate on Twitter During COVID-19

**DOI:** 10.3390/bs15020223

**Published:** 2025-02-16

**Authors:** Sushant Kumar, Shreyas Meher, Pengfei Zhang

**Affiliations:** 1 Jindal School of Government and Public Policy, O.P. Jindal Global University, Sonipat 131001, India; sushant.kumar@jgu.edu.in; 2 School of Economic, Political and Policy Sciences, The University of Texas at Dallas, Richardson, TX 75080-3021, USA; shreyas.meher@utdallas.edu

**Keywords:** COVID-19, universal healthcare, Medicare-For-All, Twitter, PNHP, P4AHCF, interest group, text analysis

## Abstract

COVID-19 has reinvigorated the policy debate for a universal healthcare system, attracting much attention on social media. In this paper, we study the online discourse of Medicare-For-All before and after COVID-19 by examining the Twitter feeds of two opposing health advocacy groups—Physicians for a National Health Program (PNHP) and Partnership for America’s Healthcare Future (P4AHCF). Our empirical results show a sharp contrast between the two interest groups’ communication strategies. PNHP showed a consistent narrative before and after the onset of COVID-19 on 11 March 2020, marked by personalized stories, references to diverse demographic groups, and a growing number of Medicare-For-All tweets. In contrast, P4AHCF showed more scientific terminology and data-centric tweets and had an inconsistent narrative with a sudden surge in positive sentiments and a complete silence on Medicare-For-All right after 11 March. The difference in communication strategies is consequential. PNHP has higher engagement with Twitter users and is more adaptive to a pandemic narrative than P4AHCF. We discuss how distinctive social media strategies can be explained by the groups’ different audiences and resources. The findings add to our understanding of healthcare advocacy campaigns on social media and the implication of a pandemic for health policy reform.

## 1. Introduction

Many have been baffled by the question: why was the U.S. hit so hard by the COVID-19 pandemic? Apart from claiming the lives of more than 1.1 million Americans so far and counting (Centers for Disease Control and Prevention, 2023), the economic crisis generated by the pandemic resulted in more than 12 million people losing employer-based health insurance at the peak of the pandemic, according to some estimates (Nova, 2020), which to critics exposed the fundamental weakness of the US healthcare system. Although there is no definite answer, it is common to hear responses like “it is a mess of a system” and “coordination in the system is so poor”. In the middle of the pandemic, we witnessed the specter of states and hospitals bidding against each other, and against the federal government, for crucial supplies for their frontline workers (Feiner, 2020). The fragmented nature of the U.S. healthcare system and lack of coordination between its various parts brought to the surface longstanding and systemic challenges to effective medical responses needed in a time like the pandemic crisis (King, 2020).

This resurfacing of systemic issues in the U.S. healthcare system reinvigorated calls for universal healthcare, with the pandemic shifting public opinion in favor of a Medicare-For-All system (Galvani et al., 2020; McDonnell, 2020). According to Morning Consult/Politico poll data, public support for Medicare-For-All went from 50% to 59% between mid-February and the end of March 2020, the highest level of support in about nine months (Murad, 2020). Perhaps not surprisingly, most countries with a universal healthcare system implemented such system in the wake of a major crisis: the U.K, France and Japan carried this out after World War II, Rwanda after the genocide, and Mexico after democratization (McDonnell, 2020).

It is in this context that it is useful to examine how COVID-19 affected the present-day discourse on universal healthcare in the U.S. To study that, the question that animates this study is how opposing health advocacy groups defined and framed the idea of a Medicare-For-All/single-payer system, the more popular universal healthcare models in the present time, with the onset of an extraordinary health crisis generated by the pandemic. To pursue this question, we examined the Twitter feeds of two leading—and opposing—health advocacy groups to trace how they frame the ideas of Medicare-For-All and single-payer.

Twitter-based public health research is a recent but growing field. The platform acts as a unique big data source based on real time content that has proven useful for disease and behavior prediction, surveillance of trends, understanding sentiments about public health issues, and engagement with health campaigns, among others (Sinnenberg et al., 2017). In the context of COVID-19 specifically, studies have found Twitter to be a useful communication channel to understand both public concerns and public awareness (Boon-Itt & Skunkan, 2020; Aiello et al., 2021). Twitter and the rise in social media, in general, has shifted the dynamics of top-down agenda setting by traditional media to more power in the hands of the public. Studies have found a symbiotic relationship between Twitter and traditional media in informing each other’s agenda (Groshek & Groshek, 2013; Conway et al., 2015). Among social media, Twitter is especially relevant to understanding agenda building because journalists tend to be heavy users of Twitter and receive story ideas and sources from it routinely (O’Connor, 2009; Farhi, 2009). Existing literature studying political discourses on health during COVID-19 using Twitter data has explored various topics including analyses of pandemic discourses (Wicke & Bolognesi, 2020), attitudes towards COVID-19 vaccination (Greyling & Rossouw, 2022; Marcec & Likic, 2022), polarization in online discourses (Mønsted & Lehmann, 2022), information gaps for communities (Caddy et al., 2023), misinformation (Broniatowski et al., 2022). But none of these papers investigate the dramatic effects of external events of the magnitude of COVID-19 on the communication strategy of special interest groups, which our paper attempts to explore.

In this context, on the one side of interest group discourse considered here is Physicians for a National Health Program (PNHP), a single-issue organization with more than 20,000 members and chapters across the U.S. PNHP has advocated for a universal, comprehensive single-payer national health program since 1987. It is also the only national-level physician’s organization of its kind. Its members and staff conduct original research on health reforms, publish peer-reviewed articles in journals, participate in town hall meetings and debates, and appear regularly in national media to advocate for a single-payer system (Physicians for a National Health Program, 2019). Since PNHP represents organized voices from within the healthcare industry (doctors) who are dedicatedly in favor of Medicare-For-All, we found it fitting to represent this discourse in our analysis.

On the opposite side is Partnership for America’s Healthcare Future (P4AHCF), an alliance of doctors, nurses, community hospitals, insurance providers, biopharmaceutical companies formed in Spring 2018. P4AHCF declares its mission as expanding access, protecting patient choice, lowering cost, improving quality and fostering innovation while opposing any “one-size-fits-all” approach to health reforms, notably Medicare-For-All, Medicare buy-in, or the public Option. Its key members include America’s Health Insurance Plans (AHIP), the Federation of American Hospitals (FAH), American Hospital Association (AHA), Blue Cross Blue Shield Association, Pharmaceutical Research and Manufacturers of America (PhRMA), and various Chambers of Commerce (Partnership for America’s Health Care Future, n.d.). P4AHCF members spent a combined $143 million on lobbying in 2018 alone (Evers-Hillstrom, 2019). Since it reflects the negative views on universal healthcare held by a broad range of organized healthcare industry stakeholders, we found it to be a particularly compelling candidate to represent the opposing side of the Medicare-For-All discourse.

To understand the differences in the communication approach of the two groups on Twitter, we employ graphical visualization and difference-in-difference (DID) analysis on the tweets of the groups. Our empirical results show a sharp contrast in how the two interest groups communicate on social media. PNHP shows a relatively consistent narrative with an increasingly higher number of tweets about Medicare-For-All after the onset of COVID-19, consistent appeal to a wide demographic base and personal stories, and relatively stable levels of references to scientific arguments and sentiment score. By contrast, in the light of the pandemic, P4AHCF is silent about Medicare-For-All, spiking the use of numbers, scientific studies, and evidence in their narrative, becoming overly optimistic in terms of sentiments, at the same time avoiding engagement with a wider demographic base or references to people’s experiences and stories.

As a result of the communication strategies, P4AHCF’s content does not have much traction among people compared to PNHP, as shown in our findings about engagement. This resulted from multiple issues in their strategy: using impersonal content as explained in the earlier paragraph, which has been found in communication research responsible for less engagement; not acknowledging issues facing people during a health emergency and sounding delusionally positive; not speaking to a wider audience by failing to highlight broader social dimensions of the problem; and using repetitive and robotic content. Their strategy clearly has hurt their engagement and connection with the voters.

The remainder of the paper proceeds as follows. Section 2 briefly reviews the background of Single-payer and Medicare-For-All in American politics in the last fifty years. Section 3 describes our hypotheses, our methodology, and the Twitter sample. Section 4 presents our text analysis results. Section 5 discusses related literature and our contribution. Section 6 concludes.

## 2. Issue Background

The modern-day idea of a single-payer healthcare system was first proposed in 1971 by Senator Edward Kennedy and Martha Griffiths to create a Canadian-inspired single-payer system, which was to be financed by one single insurer- the government, for all the medical services (Rochefort & Donnelly, 2008). However, the rightward shift in American politics in the 1970s amidst the Vietnam War, Watergate and rising anti-tax sentiment and the conservative era of Regan administration in the 1980s led to prominence of market-based solutions in the American healthcare system (Oberlander, 2019). In these situations, as Congress turned away from the issue, activists took charge of healthcare reform leading to the creation of PNHP in late 1980s, which started using the term “national health insurance” as describing their goal but soon moved to “single-payer” as their phrase of choice (Abrams, 2019). However, the idea of single-payer could not catch up beyond the health policy and activist circles and the Clinton Administration in the 1990s, distancing itself from the idea, took a more moderate approach and sought to expand coverage to everyone while keeping the role of private and employer insurance intact going for the idea of “managed competition”, signifying a rightward shift in the Democratic Party at the time (Oberlander, 2019).

The new millennium brought in a change from the technical language of “single-payer” to a more aspirational idea of expanding a domestic, well-known and functioning policy of Medicare to cover everyone, starting from Rep Jon Conyer’s Expanded and Improved Medicare-For-All Act of 2003, followed by Kennedy’s 2006 Medicare-For-All Act (Oberlander, 2019). Barack Obama initially supported the idea of “public option” but the Affordable Care Act, although being the biggest health reform since 1965, settled for something much more moderate, and it was criticized from both the left and the right. The main proponent of this model has been Vermont Senator Bernie Sanders, who has put forth five different versions of Medicare-For-All so far, with increasing support from his colleagues, reflected in an increase in the number of cosponsors for his bills from zero in 2013 to 14 for the most recent version, which included four other Democratic Presidential aspirants (Martin & Goodnough, 2019; Stein, 2018).

However, a recent poll by Kaiser Permanente suggests that there is confusion among the public about what Medicare-For-All means, with 56% supporting a true Medicare-For-All but 74% supporting a plan that gives people the option of choosing between their private insurance and governmental insurance (Kaiser Family Foundation, 2020). There are also differences in pathways suggested by Democratic politicians to achieve universal healthcare with most assuming some role of private health insurance alongside a government plan. President Biden’s support of a public option further puts the future of a Medicare-For-All on the back burner for the time being. The development of Medicare-For-All debate over the years suggests that there takes place a rightwards shift in its narrative around the time of general elections as various powerful interests align to set the parameters for what’s politically feasible. It would be worthwhile to see how the COVID-19 pandemic shifts this debate as demands for a new social contract in the form of a stronger welfare state and strengthened public health system arise.

## 3. Materials and Methods

### 3.1. Hypotheses and Theoretical Background

Our main research question for this paper is—how did COVID-19 change the narrative strategies of the two advocacy groups analyzed in this study regarding the Medicare-For-All healthcare system? To this end, we develop five main hypotheses. Our hypotheses are based on a rational decision-making framework of special interest groups (Grossman & Helpman, 2001), which connects to the theory of strategic communication (Bennett & Manheim, 2000), the theory of connective action (Bennett & Segerberg, 2013), and the theory of scientization (Krick et al., 2019).

The theory of strategic communication posits that political actors, including elites, corporations, and advocacy groups, strategically frame the policy issues in their favor to influence public knowledge, beliefs, beliefs, and actions, to achieve specific outcomes (Bennett & Manheim, 2000; Grossman & Helpman, 2001; Blumler, 2015). P4AHCF and PNHP clearly have distinct audiences and objectives regarding Medicare-For-All. P4AHCF is what is termed a protective group, whose membership is oftentimes exclusive and restricted to the section of society whose private interests align. Such protective groups work to protect the financial interests of members. P4AHCF’s financial resources are contributed by its members, prominent among which are hospital organizations, insurance companies and pharma industry, broadly representing the medical–industrial complex.

PNHP, on the other hand, is an example of a promotional group, which is invariably and explicitly non-partisan and represents a segment of society whose focus is on promoting a particularly appealing cause or value. This makes the promotional group much more inclusive (compared to a protective group) as its primary purpose is to establish wide popular support for its cause and gain as much voters’ attention. PNHP includes voices from physicians, medical students, and health professionals that support a universal single-payer national health insurance program. Medicare-For-All or the idea of universal healthcare is an intense political issue, which requires redistribution of resources to ensure that everyone in society receives access to quality care irrespective of their identity and means. PNHP works to advocate that idea, which is of broader interest of the public than of direct benefit to its members.

In our first two hypotheses, we conjecture how COVID-19 might change the two groups’ communication strategies because of their divergent objectives. For PNHP, since it is a promotional group, we posit that the group would leverage COVID-19 to double down on their advocacy for Medicare-For-All.

**H1:**  *Compared to the pre-COVID level, PNHP increases the proportion of tweets on Medicare-For-All and becomes more engaging.*

For P4AHCF, we posit that the group opposing reforms would try to avoid discussion of Medicare-For-All in the light of COVID-19, which exposed the weaknesses of the American healthcare system, as it would put pressure on the medical–industrial complex for reforms.

**H2:**  *Compared to the pre-COVID level, P4AHCF decreases the proportion of tweets on Medicare-For-All and becomes less engaging.*

In our next three hypotheses, we contrast the communication strategies of the two interest groups. As predicted by the theory of strategic communication, the distinct audiences and objectives would determine the two groups’ different communication strategies (Bennett & Manheim, 2000; Grossman & Helpman, 2001). As a promotional group, PNHP’s objective is to win the support of the public, especially the voters. Therefore, individual stories and users’ endorsement are the optimal strategy for them. PNHP refers to stories and experiences of the public to increase the proximity of its narrative and make it more relatable to the public to create a more convincing case in favor of a Medicare-For-All or single-payer system. It tweets: “Charlie woke up from her nap and gave me a huge hug for pushing #Medicare-For-All. Charlie was a micro preemie, just like my own kid. They are miracles. The costs of Charlie’s health care has made her mom, Rebecca, a super advocate for #Medicare-For-All. Thank you Rebecca & Charlie!” (@PNHP:2019-04-30). This also allows PNHP to speak to a wider audience. PNHP also leverages temporal issues such as the Black Lives Matter movement to expand its reach and invite new stakeholders in its audience, showing successfully in its engagement.

This is consistent with the logic of connective action proposed by Bennett and Segerberg (2013). The theory postulates that because personally expressive content is better able to be shared and recognized by others, they are more likely to propagate through social media. The resulting networks can have the potential to bypass collective action problems and provide opportunities for “connective action” that attract and engage the previously unvoiced (Blumler, 2015). We conjecture that prior to COVID-19, PNHP, the group advocating for healthcare reforms, would try to build a broad-based coalition for its cause to invite a wider audience in the discussion for healthcare reforms.

**H3:**  *PNHP’s tweets have more references to demographic groups and personalized stories than P4AHCF.*

In contrast, P4AHCF would try to minimize the participation of a wider audience in the discussion, scientize the conversation, and find recluse in data and statistics, making it more esoteric to maintain the status quo and avert calls for universal healthcare.

Scientization refers to the growing reliance on scientific expertise to back up political claims and come up with viable policy solutions (Krick et al., 2019). Apart from the states’ attempt to leverage science and appear as a “rational actor”, advocacy groups also use robust scientific arguments to bolster their claims (Yearley, 2005). Scientization of policy issues helps depoliticize them and make political actors appear more reliable in the eyes of citizens (Marcussen, 2011).

As a protectionist group, P4AHCF’s objective is to maximize the payoff of its group members—hospital management boards, insurance plans, and various Chambers of Commerce. With substantial lobbying spending, their focus is to maintain the status quo with the policymakers in the White House. The data-centric strategy “scientized” the healthcare discussion and took it away from popular scrutiny and into the domain of technical expertise, which cannot be questioned easily by the public, thus, disengaging from popular discontent about the state of healthcare amid a pandemic. This databased strategy also speaks to the policymaking community, which relies way more on “data” in this era marked by “evidence-based” policymaking. This is an optimal strategy for P4AHCF because their tweets are a statement of their policy stance, possibly for future legislative hearings.

**H4:**  *P4AHCF’s tweets have more statistics and scientific reports than PNHP.*

In contrast to numbers and statistics, individual stories and experience would naturally have a better resonance with the public (Bennett & Segerberg, 2013). As a result, PNHP’s narrative would be much more engaging than P4AHCF.

**H5:**  *PNHP’s tweets have higher engagement than P4AHCF.*

### 3.2. Research Design

Our main method is difference-in-difference (DID) analysis and graphical visualizations of the two groups’ social media behaviors based on Twitter data (Conway et al., 2015). The data for the PNHP account start from April 2017 and for the P4AHCF account from June 2018, when the P4 account was created. These dates were based on the extent to which Twitter API allowed one to go back in order to extract the data; in case of P4, it was limited by the non-existence of the account before June 2018. We identified and extracted all tweets for the PNHP account from April 2017 to May 2023 and all tweets for the P4AHCF account from June 2018 to July 2023. In total, we collected 3659 tweets for the PNHP account and 1381 tweets for the P4AHCF account. For our statistical analysis, we aggregate these tweets by month, smoothing out temporal variations on the daily or weekly basis.

All statistical analyses were performed using R version 4.4.2 “Pile of Leaves” (R Core Team, Boston, MA, USA; https://cran.rstudio.com/, accessed on 14 January 2024). The code and data used in this analysis are available in the Harvard Dataverse repository (https://dataverse.harvard.edu/dataset.xhtml?persistentId=doi:10.7910/DVN/OWVD70, accessed on 14 January 2024). Using the methodology described above, we assembled a monthly panel dataset of tweets from the two interest groups. For each tweet, the following information is recorded: timestamp, text, hashtags, retweets, favorites, users mentioned. The hashtags include #Medicare-For-All, #SinglePayer, #COVID19, #WorkingTogether, #VoterVitals, etc. When performing a comparison, we also calculate the proportion of “Medicare-For-All” tweets (monthly counts divided by the total number of tweets per month), the mention of different demographics groups in the tweets, the proportion of tweets that include scientific key words, and the sentiment scores. In identifying and counting mentions of “Medicare-For-All”, we use both hashtags and tweet text, where we employ regular expressions to detect various forms of the phrase, accounting for case sensitivity and different spellings.

To identify the causal effects of COVID-19 on interest groups’ communication strategy, we apply a difference-in-difference design for credible estimates. Let i be an interest group and t be a calendar month. For different outcome variables, Y_it_, we estimate the following regression:

Y_it_ = β_0_ + β_1_Group_i_ + β_2_Post-Covid_t_ + β_3_ Group_i_ × Post-Covid_t_ + ε_it_ (1)

where Group_i_ = 1 if the group is PNHP, and 0 for the baseline group of P4AHCF. Post-Covid_t_ = 1 after the treatment date is 11 March 2020, when the WHO declared COVID-19 a pandemic. The coefficient β_1_ measures the pre-existing differences in communication strategy between the two groups. The coefficient β_2_ measures the pre-post difference in outcome of interests over time in relation to COVID-19. The coefficient β_3_ measures the effects of COVID-19 on the changes in communication strategy. The estimation of β_3_ tests the hypotheses H1 and H2: we would expect β_3_ to be positive for proportion of Medicare-For-All tweets and engagement. The estimation of β_1_ tests hypotheses H3, H4, and H5: we would expect it to be positive for proportion of demographics-related tweets, negative for scientization tweets, and positive for engagement.

Notice that our DID model is a modified version of a conventional DID design where one group is treated and the other is not. In our case, both groups are treated by the pandemic shock of COVID-19, but because of the difference in the underlying strategies, the effect of the pandemic goes almost in opposite directions for the two groups. In this sense, we can view this model as testing heterogeneous effects of the pandemic on communication strategies. The estimation of β_3_ improves upon a correlation exercise in that it allows us to rule out concerns that could otherwise undermine a causal interpretation, including the pre-existing differences between the groups, and common trends in social media communication over time.

**Figure 1:**
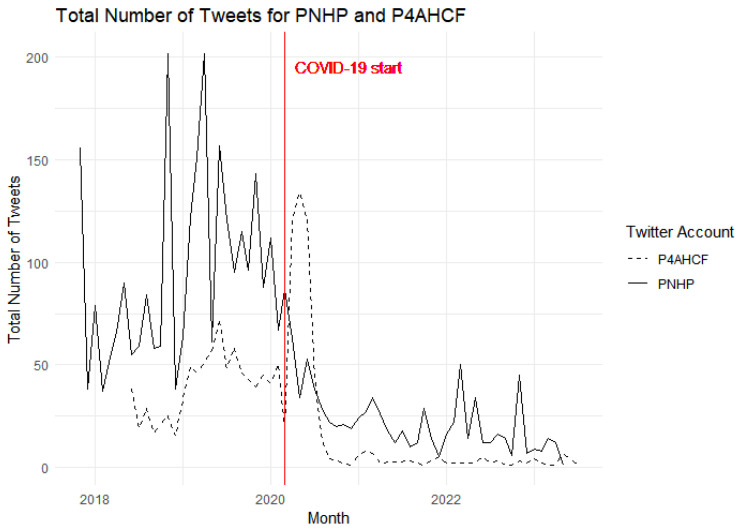
Total Number of Tweets across time.

Our graphical visualization allows us to observe trends and changes over time, particularly around key events such as the onset of the COVID-19 pandemic. Figure 1 presents a comparative analysis of the monthly tweet volumes for two Twitter accounts, PNHP and P4AHCF, over a multi-year period. The solid line represents PNHP, which demonstrates variable activity with several peaks, and the dashed line represents P4AHCF, which generally indicates a lower level of tweeting activity. A red vertical line, labeled “COVID-19 start”, intersects the timeline in March 2020, providing a time reference for the onset of the global pandemic.

At the outset of COVID-19, a substantial spike in activity is observed for P4AHCF, potentially reflecting an intensified engagement with pandemic-related topics or a response to heightened public interest during this period. In contrast, PNHP’s activity, while higher overall, does not exhibit a similar sharp increase; instead, it shows a slight decline followed by a gradual recovery to pre-pandemic levels. This contrast may suggest differing strategies or focuses of the two groups in response to the pandemic, with P4AHCF possibly capitalizing on a more focused set of topics that gained relevance during the early stages of COVID-19.

The Summary Statistics in Table 1 presents the summary statistics of our sample. It is evident that PNHP shows a significant increase in the proportion of tweets mentioning “Medicare-For-All” post COVID-19 compared to the pre-COVID-19 period. Specifically, PNHP’s “Medicare-For-All” proportion increased from 50.1% to 85.5%, indicating a strong focus on this topic after the onset of the pandemic. In contrast, P4AHCF’s proportion of “Medicare-For-All” has dropped from 40.3% to 4.6%. Moreover, while both accounts saw a decrease in overall tweet counts, PNHP’s drop was less pronounced in terms of maintaining focus on “Medicare-For-All” topics. This demonstrates that PNHP not only maintained but significantly increased their engagement and reference to “Medicare-For-All” post COVID-19. In terms of scientization tweets, both accounts experienced a decrease in the proportion of such tweets post COVID-19. PNHP’s average word count remained relatively stable, whereas P4AHCF saw a slight reduction. Additionally, PNHP’s normalized retweets and favorites (calculated as the total retweets and favorites divided by the total number of tweets) remained relatively high post COVID-19, indicating sustained engagement despite the overall decrease in activity.

## 4. Results and Discussion

### 4.1. Medicare-For-All Mentions

**Figure 2:**
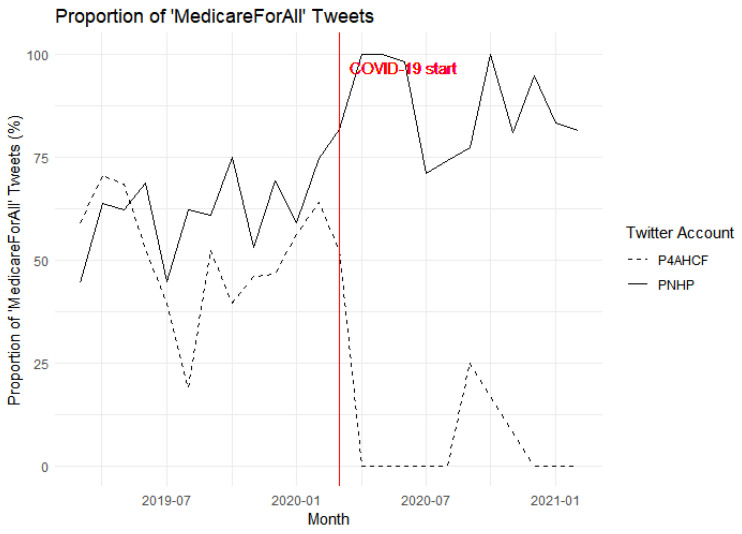
Proportion of “Medicare-For-All” Tweets.

Figure 2 illustrates the impact of COVID-19 on the narratives of the two groups based on their tweets on Medicare-For-All/Single-payer before and after 11 March 2020. The solid line representing PNHP shows fluctuations in the proportion of topic-specific tweets, with a noticeable increase leading up to the onset of COVID-19, marked by the red vertical line. Consistent with our first hypothesis, H1, this suggests an escalation in discussions around “Medicare-For-All” by PNHP as the pandemic began, possibly a reflection of the growing discourse on healthcare issues spurred by the crisis. Conversely, the dashed line for P4AHCF reveals a different pattern, with proportions generally lower than PNHP’s before the pandemic and a sharp decline to zero immediately after the pandemic starts. This drop indicates that P4AHCF shifted focus away from tweeting about “Medicare-For-All” after the onset of the pandemic, which provides suggestive evidence for our second hypothesis, H2.

To statistically test H1 and H2, Column 2 in Table 2 reports the difference-in-difference estimate of the effect of COVID-19 on the proportion of tweets. COVID-19 significantly increases the proportion of tweets discussing “Medicare-For-All” for PNHP as opposed to P4AHCF. The estimates tell us that, holding all other factors constant, COVID-19 led to a 50.706 percentage point more in the PNHP’s “Medicare-For-All” tweets, and this effect is precisely estimated (*p* < 0.005). To put the estimates in context, PNHP nearly doubled their efforts in advocating universal healthcare as a response to COVID-19, representing a substantial shift towards an intensified discussion on “Medicare-For-All”. PNHP clearly sees COVID-19 as an opportunity to push for reform considering the ongoing healthcare debates.

**Figure 3:**
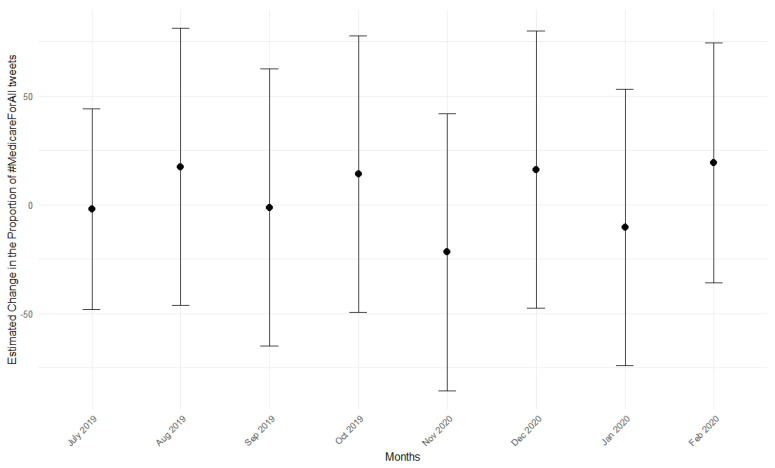
Pre−Trends Event Plot for “Medicare-For-All” Mentions.

Figure 3 presents the event study graph of the effect on the proportion of Medicare-For-All tweets. The coefficients on pre-treatment leads are nearly zero, and their standard errors are small. There was no difference between the trending tendencies of the two groups prior to COVID-19, validating the parallel trend assumption.

### 4.2. References to Demographic Groups

PNHP is engaging in the strategy of creating a broad-based coalition for its cause, by alluding to the issues of race, income status, age, gender (marginally) and immigrant communities while advocating for Medicare-For-All (See Table 3 below for the schema used to code for the themes of race, gender, low-income, age and immigration status). PNHP tweets: “Our health care crisis is a racial justice issue, 59% of the uninsured are people of color. #Medicare-For-All” (@PNHP:2019-10-29). “We should remember that, even if every state expanded Medicaid, millions of immigrants would remain uninsured…” (PNHP:2019-10-29). “Low-income workers and their families are falling through the cracks of our fragmented, dysfunctional health care financing system. #SinglePayer #Medicare-For-All would improve coverage for these workers, and for everybody else” (@PNHP:2019-12-05).

For its part, P4AHCF makes zero or minimal reference to race, gender, income, or immigrant status of those experiencing obstacles to coverage in the current healthcare system while talking about Medicare-For-All and single-payer system. When it does refer to income status, it does so in connection with how middle-income families stand to lose if a Medicare-For-All system is adopted. It tweets: “Our latest #VoterVitals poll finds voters top health care priority is lowering costs, but a one-size-fits-all new government insurance system like Medicare-For-All would raise taxes on middle class families. We can’t afford a one-size-fits-all system” (@P4AHCF:2020-03-11).

**Figure 4:**
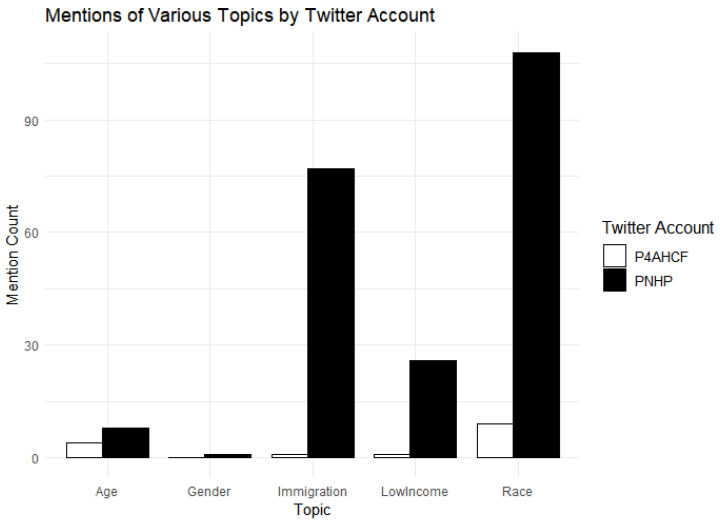
Overall mention of different demographic groups.

**Figure 5:**
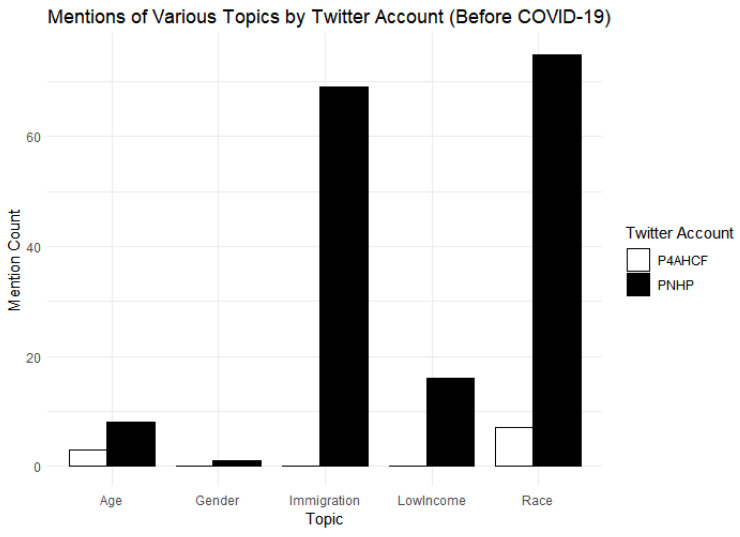
Mention of demographic groups before COVID-19.

PNHP’s references to a variety of demographic groups continued before as well as after the onset of COVID-19 (See Figure 4 and Figure 5) in contrast to P4AHCF, which had minimal references to these themes. PNHP also leveraged the larger economic crisis prompted by the pandemic, leading to large-scale unemployment and loss of health insurance for millions of Americans, to point to the pitfalls of the employer-sponsored coverage, to push for healthcare as a “right” and to promote Medicare-For-All and Single-payer system as a remedy. Highlighting these aspects, PNHP tweets: “This #COVID19 crisis proves we need health care as a human right, not an employment benefit”. #SinglePayer #Medicare-For-All” (@PNHP:2020-06-30). Similarly, PNHP used the suddenly salient racial justice movement to point out racial disparities in the U.S. healthcare system and how it could be addressed by Medicare-For-All/Single Payer. It tweets: “How has slavery’s legacy impacted present day health disparities? How does systemic racism perpetuate these health disparities? How can a #Medicare-For-All system begin to address racial health inequities?” (@PNHP:2020-07-01).

In contrast, consistent with the strategy of minimizing the scope of discussion, P4AHCF makes minimal reference to race, income or immigrant status of people in its tweets, even avoiding talking about the racial issues in the American healthcare system in the light of the Black Lives Matter movement. Furthermore, the narrative offered by P4AHCF shifted after March 11 with the onset of COVID-19. It started focusing instead on firefighting the issues arising within the healthcare system due to the COVID-19 and its economic impacts in a way of putting the house in order or cleaning up its act. A new theme of #Workingtogether emerged frequently in its tweets in this period, emphasizing how different industry stakeholders, including healthcare providers, hospitals, insurance industry and pharmaceutical companies, were working together to defeat COVID-19, expand coverage, and provide Americans with adequate healthcare. It tweets: “What do America’s leading doctors, nurses, clinicians, hospitals, health insurance providers, biopharmaceutical companies and employers all have in common? They’re #WorkingTogether to ensure Americans get healthy and stay healthy” (@P4AHCF:2020-04-15). Further, it made sure to highlight the merits of the free market in ensuring patient choice, freedom and quality even during a pandemic by tweeting: “The free market is #WorkingTogether to keep control in the hands of patients as they choose where and how they receive care during the #COVID19 crisis” (@P4AHCF:2020-05-18).

Table 2 also reports the difference-in-difference estimate of the effect of COVID-19 on the proportion of topic-specific tweets. The pre-existing difference between the groups is significant (*p* < 0.001) with PNHP having 4.3598 percentage points higher on the proportion of tweets discussing demographics-related topics. There is a notable emphasis on specific demographic groups by the PNHP compared to P4AHCF, and that difference persists throughout the pandemic. This result supports Hypothesis H3. The treatment effect of COVID-19 in this case, however, is not statistically significant (*p* = 0.560), suggesting that there was no significant change in the proportion of topic-specific tweets post COVID-19 between the two groups.

### 4.3. Scientization

**Figure 6:**
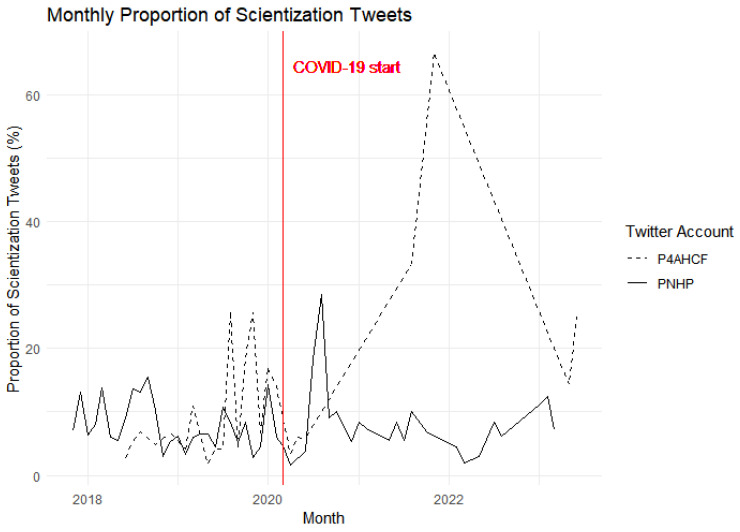
Scientization tweets across time.

Next, we examine the extent to which the two groups chosen here scientize the idea of Medicare-For-All. Figure 6 depicts the monthly total of ‘scientization’ tweets from the PNHP and P4AHCF Twitter accounts, covering the period of one year before and after the declaration of the COVID-19 pandemic, indicated by the prominent red vertical line. The scientization-related keywords used to filter and create this plot were “numbers”, “scien\w*”, “study”, “evidence”, “evidence-based”, “statistics”, “data”, “research”, “analysis”, “findings”, “clinical”, “scientific”, “data-driven”, “experiment”, and “quantitative”, totaling fifteen keywords. These terms were selected to capture the essence of scientific discussion and evidence-based dialog within the tweets of these two accounts, reflecting on how their communication strategies might have been influenced by the evolving public health crisis. We used this as a proxy to understand the extent to which both groups scientize the conversation about Medicare-For-All reforms.

After the onset of COVID-19, the P4AHCF exhibits a significant surge in “scientization” tweets at the juncture labeled “COVID-19 start”, whereas PNHP shows a relatively low and constant level of activity. The conspicuous increase for P4AHCF suggests a heightened focus on scientific discourse as the pandemic unfolds, potentially signaling a strategic emphasis on data-driven response to public concern regarding the health crisis. In contrast, the steady pattern observed for PNHP may reflect an already established, consistent engagement with scientific topics, undisturbed by the onset of the pandemic. This is consistent with Hypothesis H4. This divergence in tweeting behavior could imply differing organizational priorities or audience engagement strategies during a period marked by increased public attention to scientific and health-related information.

Table 2 reports the difference-in-difference estimate of the effect of COVID-19 on the frequency of scientization tweets. The pre-existing difference between the two groups is significant (*p* < 0.008) with PNHP having 2.645 percentage points lower on the proportion of scientization tweets, supporting Hypothesis H4. COVID-19 widened this gap and further reduced the proportion of scientization tweets by PNHP as opposed to P4AHCF. The estimates tell us that, holding all other factors constant, COVID-19 led to a 4.117 percentage point decrease in the proportion of scientization tweets (*p* = 0.016). In other words, P4AHCF were more likely to justify their policy stances and neutralize the debate on universal healthcare by references to technical statistics and numbers, as COVID-19 did not work in their favor.

### 4.4. Sentiment Analysis

**Figure 7:**
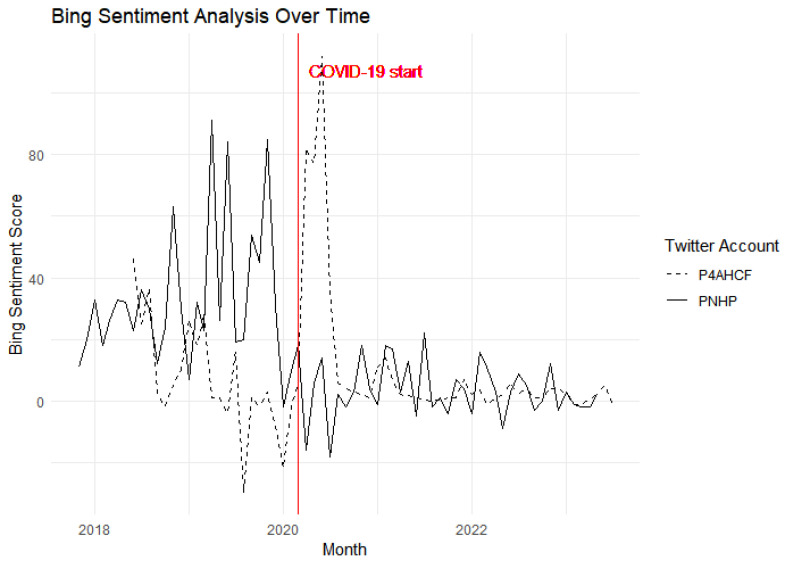
Bing Sentiment Scores across time.

Figure 7 showcases a Bing sentiment analysis over time for tweets from the PNHP and P4AHCF accounts. A Bing sentiment score is a binary score that indicates whether a word is positive or negative. The sentiment score for a text is the sum of the individual word scores and is calculated using the Bing lexicon, which categorizes words into positive or negative sentiments, and then aggregates these values for a net sentiment score. The sentiment analysis shows a noticeable surge in sentiment score for P4AHCF at the start of the pandemic. This upward spike, depicted by the dashed line, could indicate an increase in positive messaging or a concerted effort to engage with the audience on a more positive note during the uncertain times marked by the beginning of the pandemic.

In contrast, the sentiment trajectory for PNHP, represented by the solid line, remains relatively stable without dramatic shifts, suggesting a consistent tone in their Twitter communications throughout the same period. The marked divergence in sentiment response between the two accounts at the onset of COVID-19 is particularly striking. P4AHCF’s pronounced sentiment increase could reflect a strategic pivot in their narrative to address the pandemic’s challenges.

At first glance, the upswing in positive sentiment for P4AHCF during a period typically associated with uncertainty and anxiety seems counterintuitive. This anomaly prompts a deeper inquiry into the nature of the communication strategies employed by P4AHCF during the emergent phase of the pandemic. One could speculate that P4AHCF’s communications may have strategically focused on fostering a sense of agency and collective resilience. It is conceivable that their tweets during this period were imbued with constructive narratives, emphasizing actionable insights, scientific advancements, and community solidarity—all of which could be coded as positive by the sentiment analysis algorithm. Fittingly, the prevalence of terms typically associated with progress, like “innovation” and “research”, in P4AHCF’s tweets suggests that its communication strategy may have been heavily oriented towards positive, forward-looking messages. Such an approach would not only diverge from the prevailing tone of discourse at the time but also position P4AHCF as a source of proactive guidance amidst the burgeoning crisis.

Table 4 below reports the difference-in-difference estimate of the effect of treatment on Bing sentiment scores. The baseline model shows that the effect of the treatment (PNHP as the treatment group and P4AHCF as the control group) is significantly negative following the intervention. The treatment, adjusted for both time and unit fixed effects, decreases the Bing sentiment scores. This estimated effect is robust to the inclusion of unit-fixed effects, year-fixed effects, and time-variant control variables. The estimates tell us that, holding all other factors constant, the treatment led to a 33.831-point decrease in Bing sentiment scores, and this effect is statistically significant (*p* = 0.0000124). To put the estimates in context, the treatment effect size is considerable, reflecting a significant downturn in sentiment scores for PNHP compared to the control group (P4AHCF), influenced by the COVID-19 pandemic.

We also complement the Bing sentiment analysis with two other methods—AFINN and NRC—presented in the Appendix A. AFINN assigns a numeric value to each word for its sentiment strength, while NRC classifies words into emotional categories including positive and negative sentiments. The consistency of results across these diverse sentiment analysis methods strengthens the robustness of the findings. An aggregated sentiment score, combining insights from all three methods, offers a comprehensive view of the overall sentiment trends. This multi-faceted approach ensures that sentiment analysis is not reliant on a single lexicon, thereby enhancing the credibility of the conclusions drawn from the sentiment trends observed in the tweets.

### 4.5. Engagement

**Figure 8:**
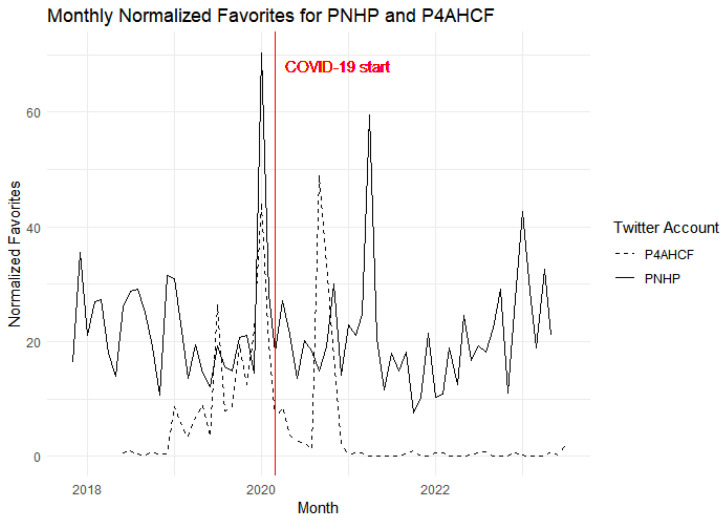
Normalized Favorites for the two groups over time.

**Figure 9:**
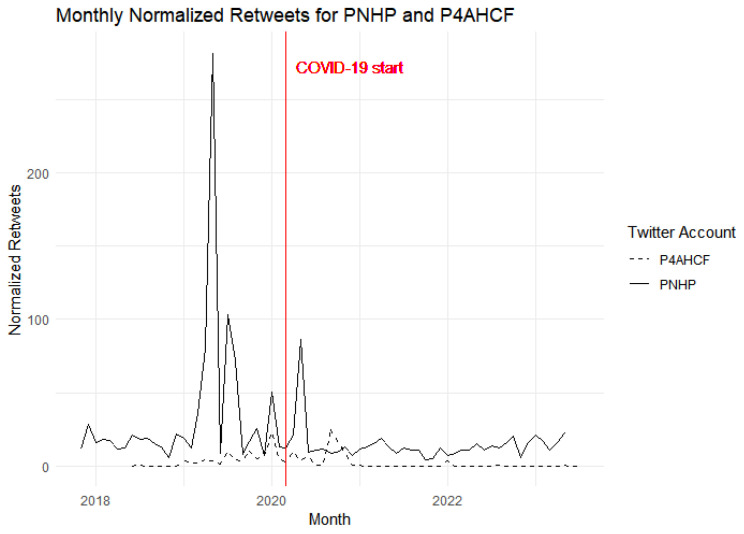
Normalized Retweets for the two groups over time.

Figure 8 and Figure 9 represent engagement with the content of both groups over the course of time. It is worth mentioning here that PNHP had 17,800 followers while P4AHCF had 23,000 followers as of 12 January 2024. Despite having a relatively smaller follower base, PNHP’s tweets show consistently higher engagement than P4AHCF both in terms of likes and retweets before and after the onset of the pandemic. Figure 9, showing the normalized retweets, echoes a similar trend to the first one showing normalized likes or favorites. PNHP experiences more pronounced peaks and sustains elevated engagement levels in comparison to P4AHCF. The normalized retweet count is an indicator of the content’s reach and the audience’s willingness to share it within their networks. PNHP’s ability to consistently achieve higher likes and retweet counts could be attributed to a variety of factors, including the relevance and relatability of their tweets or a more engaged core follower base.

The brief spike in engagement for both accounts at the beginning of the pandemic suggests a heightened public interest in health-related content during that period. However, PNHP maintains a lead over P4AHCF, which could imply that their messaging or content strategy is particularly effective in eliciting a response from their audience. It is a matter of further research whether greater engagement with PNHP’s tweets is because of its contents being more people-centric, speaking to a wider audience and leveraging contemporary issues like the Black Lives Matter movement to make its narrative more personally relatable. Similarly, it needs further investigation whether the overly scientized and optimistic narrative of P4AHCF during the pandemic fell out of sync with the public mood during a rare human tragedy leading to lesser engagement.

Table 4 reports the difference-in-difference estimate of the effect of treatment on normalized favorites and retweets, representing engagement for the purpose of this paper. The pre-existing difference between the groups is significant (*p* < 0.05) with PNHP having 13.562 percentage points higher normalized favorites and 30.4 percentage point higher normalized retweets. The baseline model, however, shows that the effect of the treatment (PNHP as the treatment group and P4AHCF as the control group) is not significant for normalized favorites (*p* = 0.1910) or normalized retweets (*p* = 0.08150), suggesting that there was no significant difference in the proportion of favorites or retweets, and hence engagement, post COVID-19 between the two groups.

### 4.6. Interaction Network

**Figure 10:**
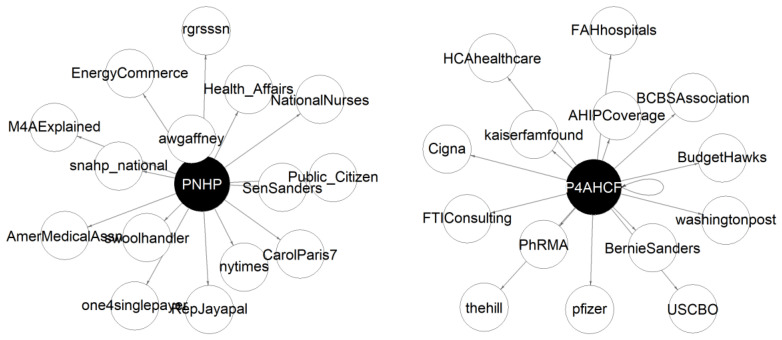
Interaction Plot for PNHP and P4AHCF.

Network analysis reveals minimal overlap in Twitter interactions between PNHP and P4AHCF, despite both organizations addressing healthcare policy issues. This separation is strikingly visible in Figure 10, which visualizes their top 15 most frequent interactions, where PNHP’s network shows frequent engagement with progressive healthcare advocacy accounts and medical professionals, while P4AHCF’s network centers on insurance companies, hospitals, and pharmaceutical companies. The comprehensive analysis of all interactions reinforces this pattern of distinct spheres of influence. Of the 997 unique accounts PNHP interacts with and 297 accounts P4AHCF engages with, only 73 accounts overlap—representing just 7.32% of PNHP’s total interactions. PNHP casts a wider net in their interactions, engaging with nearly three times as many unique accounts as P4AHCF, suggesting a more grassroots approach to advocacy compared to P4AHCF’s focused interaction pattern with industry stakeholders.

The few overlapping accounts in their networks are primarily mainstream media outlets (nytimes, washingtonpost, thehill) and key political figures (SenSanders, RepJayapal), as shown in Figure 10. However, even these shared connections reveal different engagement patterns. For instance, PNHP mentions The New York Times 66 times compared to P4AHCF’s 5 mentions, and Senator Sanders 64 times compared to P4AHCF’s 7 mentions as shown in Table 5. This analysis suggests these organizations primarily operate in distinct networks with different priorities in their Twitter engagement strategies. While they are not completely isolated from each other’s spheres of influence, as evidenced by 73 overlapping accounts across both PNHP and P4AHCF, the minimal overlap and different interaction patterns indicate these organizations are largely “talking to different audiences”, with limited cross-pollination of ideas occurring mainly through shared media and political interactions.

## 5. Contribution and Related Literature

Our paper makes three important contributions. First, it provides a timely analysis of the impact of COVID-19 on interest group narratives—an area of inquiry worthy of ex-ploration in its own right. The existing, scant literature on this topic focuses on advocacy coalition building (Raeymaeckers & Van Puyvelde, 2021), influences on social justice movements (Pleyers, 2020), and the operations of civil society organizations themselves (Nosko & Ušiak, 2023). However, none of the few papers we could access discuss the influence of the pandemic on the discursive strategies used by interest groups—a gap our paper aims to fill.

Second, our study contributes to the virtually non-existent literature on the social media narrative strategies of American advocacy groups concerning universal healthcare policy, represented here by Medicare-For-All. Existing research on this topic largely examines the history of the idea (Oberlander, 2019), strategies for its adoption (Morone, 2017), and questions of efficacy and implications (Schulman & Milstein, 2019). However, we find no literature analyzing narratives surrounding Medicare-For-All—let alone the social media narratives of American advocacy groups on this subject—an oversight our paper seeks to address.

Third, and most importantly, our research underscores the significance of social media data—particularly from Twitter—in understanding the political narratives that shape public opinion on health policy issues. Prior studies analyzing political discussions on health during COVID-19 using Twitter data have explored topics such as pandemic-related discourse (Wicke & Bolognesi, 2020), public sentiment toward COVID-19 vaccination (Greyling & Rossouw, 2022; Marcec & Likic, 2022), the polarization of online conversations (Mønsted & Lehmann, 2022), informational disparities affecting communities (Caddy et al., 2023), and the spread of misinformation (Broniatowski et al., 2022). However, none of these studies examine how large-scale external crises like COVID-19 influence the communication strategies of special interest groups concerning Medicare-For-All—a gap our research aims to fill.

## 6. Conclusions

In this paper, we explored the communication strategies of two opposing interest groups on Medicare-For-All and how it is impacted by the onset of COVID-19 pandemic. The empirical results support all five hypotheses. Consistent with the theory of strategic communication, the two advocacy groups have very different narrative strategies to push for their cause, which also responds very differently to an external shock like COVID-19. Consistent with H1 and H2, PNHP shows a sharp increase in tweets about Medicare-For-All after the onset of COVID-19, whereas P4AHCF’s tweets on the topic drop to zero immediately after the pandemic began. This difference in their Medicare-For-All discourse is also statistically significant in the DID regressions. Consistent with H3, PNHP’s tweets reference various demographic groups significantly more than that of P4AHCF in pre-COVID Medicare-For-All discussions. Consistent with H4, P4AHCF exhibits a significant surge in scientization tweets after the onset of COVID-19, whereas PNHP maintains a relatively low and constant level of engagement with data-driven language, unaffected by the pandemic. Finally, consistent with H5, we find a significant difference in engagement between the two groups before the onset of COVID-19, with PNHP receiving considerably more engagement, measured by likes and retweets. In response to big shocks like COVID-19, the P4AHCF’s data-centric strategy is far less flexible or adaptive than the PNHP’s people-centric strategy. Individual stories and people-centric content have the room to pick and choose, whereas statistics are harder to manipulate for self-serving purposes. This also explains why P4AHCF suddenly goes radio silent on Medicare-For-All after March 11.

We conclude our paper with a few limitations and promising extensions. Although Twitter has emerged as a valuable data source for analyzing public sentiment on health issues, it is not the only platform for studying online discourse on Medicare-For-All. Other social media platforms, such as Facebook and TikTok, have also become vehicles for advocacy campaigns, and exploring them could yield valuable insights. Moreover, while social media discourse provides a valuable lens for examining public discussions on policy issues—especially during a pandemic when mobility is restricted—it offers only a partial view of public health advocacy. Advocacy efforts extend beyond social media to grassroots organizations, traditional media, and legislative forums.

The conventional understanding of interest groups is that they target policymakers with lobbying spending and campaign contributions. Policymakers, however, are not the only targets of advocacy. Equally important, groups also aim their educational campaigns directly at the public (Krick et al., 2019). The voting public, even more than politicians, often lacks the in-depth knowledge of policy issues that is needed to evaluate the various policy options. The interest groups are eager to provide the public with policy-relevant information to facilitate framing of the issue in a way that is favorable to their own cause. Both PNHP and P4AHCF try to lead the public on Medicare-For-All. PNHP appears to be more successful at educating voters on social media. It is more popular, more engaging, and receives more attention.

According to the LobbyView database on congressional lobbying reports (Kim, 2018), P4AHCF has been an active lobbyist to the Congress on health issues and Medicare/Medicaid, spending 20,000 USD quarterly in the year 2019–2020. PNHP, on the other hand, has no recorded spending with K-street firms. This is consistent with our findings that PNHP focuses on steering the public opinion in favor of healthcare reforms, whereas P4AHCF pushes their stakeholders’ interests with endowed financial resources and direct lobbying. When and how health advocacy groups balance their communication strategy with the financial strategy is beyond the scope of this paper and would be interesting for future research.

Not coincidentally, the pandemic period also saw increasing public support for a greater governmental role in the healthcare system and for a national health plan, evident in the jump in public support for Medicare-For-All right after the onset of COVID-19 (Kaiser Family Foundation, 2020). The COVID-19 pandemic exposed the need for a universal healthcare system, which the opponents of reforms found best to avoid commenting on during a time when most people could personally relate to the deficiencies of the American healthcare system. The higher engagement of PNHP’s tweeting strategy, however, does not necessarily mean that the group will be more effective in shaping the actual health policy. How the political activism on social media affects the policymaking process is worth more exploration.

## Figures and Tables

**Table 1:** Summary Statistics.

Variables	P4AHCF		PNHP	
	Pre	Post	Pre	Post
Tweets	842	539	2666	905
Medicare-For-All Mentions	539	25	1335	774
Medicare-For-All Proportion	40.3%	4.6%	50%	85.5%
Scientization Tweets	71	24	191	45
Scientization Proportion	8.4%	4.4%	7.1%	4.9%
Demographic Topic Tweets	10	5	168	57
Demographic Topic Proportion	1.1%	0.9%	6.3%	6.2%
Average Word Count	30	27	30	30
Retweets	4007	2644	91,155	13,668
Favorites	9068	2317	56,854	18,334
Normalized Retweets	4.2	1.6	34.6	14.6
Normalized Favorites	9.5	2.4	23	20.8
Bing Sentiment Score	7.0	11.8	32.7	3.6

**Table 2:** DID Regressions I.

Variable	Proportion of Medicare-For-All	Scientization	Demographic Topics
Group_i_	18.136 (6.751) **	−2.6450 (0.9690) **	4.3598 (1.2075) ***
Post-COVID_T_	−20.021 (6.533) **	−0.7479 (1.4154)	−1.3275 (1.1546)
Group_i_ × Post-COVID_T_	50.706 (8.794) ***	−4.1170 (1.6703) *	−0.9067 (1.5513)
Number of Months	123	75	123
Number of Tweets	5040	334	235
Adjusted R^2^	0.56	0.2752	0.1939

Notes: Standard errors in parentheses. * *p* < 0.05, ** *p* < 0.01, *** *p* < 0.001.

**Table 3:** Keyword Dictionary.

Indicator	Keywords Used
Race	race, racism, racial discrimination, racial disparities, racial inequality, ethnic, ethnic group, ethnicity, minority
Low Income	poverty, socioeconomic, economic inequality, income gap, financial hardship, disadvantaged, impoverished, underprivileged
Gender	gender equality, gender discrimination, gender bias, gender pay gap, gender identity, sexism, gender stereotypes, LGBTQ+
Immigration	immigrants, immigration policy, undocumented, refugee, asylum seekers, border control, migration, citizenship
Age	elderly, senior citizens, aging population, ageism, generational, youth, baby boomers, millennials

**Table 4:** DID Regressions II.

Variable	Bing Sentiment	Normalized Favorites	Normalized Retweets
Group_i_	25.619 (5.752) *	13.562 (2.879) *	30.400 (7.723) *
Post-COVID_T_	4.758 (5.530)	−7.100 (2.753) *	−2.580 (7.385)
Group_i_ × Post-COVID_T_	−33.831 (7.412) ***	4.864 (3.699)	12.432 (9.922)
Number of Months	123	123	123
Number of Tweets	5040	5040	5040
Adjusted R^2^	0.1939	0.2242	0.4245

Notes: Standard errors in parentheses. * *p* < 0.05, *** *p* < 0.001.

**Table 5:** Top 4 Common Accounts Mentioned by Both Organizations.

Account	PNHP Mentions	P4AHCF Mentions
nytimes	66	5
SenSanders	64	7
RepJayapal	52	4
EnergyCommerce	44	8

**Figure A1:**
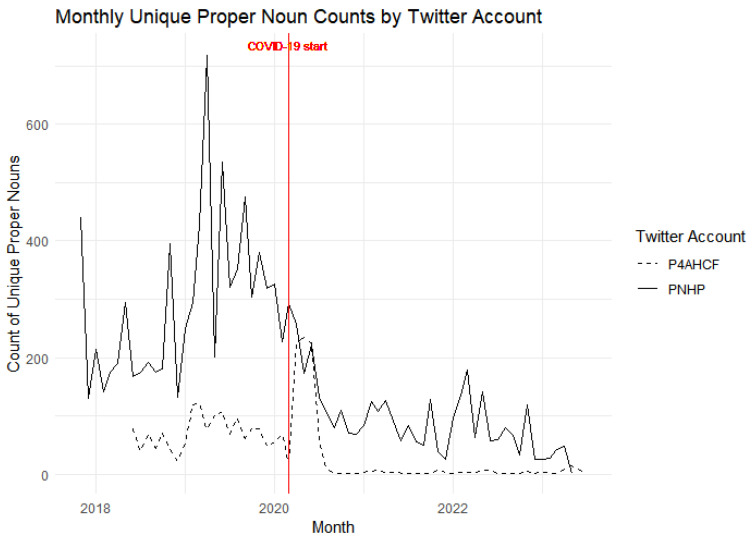
Monthly Unique Proper Noun Counts.

**Figure A2:**
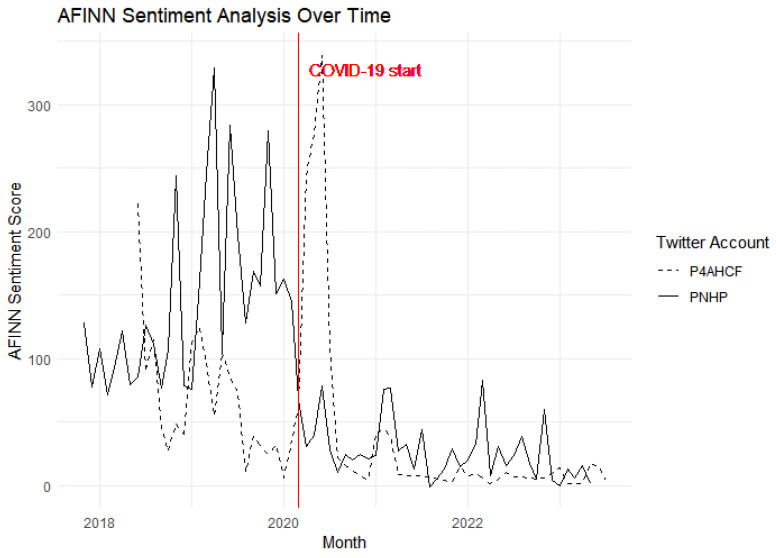
AFINN Sentiment Scores across time.

**Figure A3:**
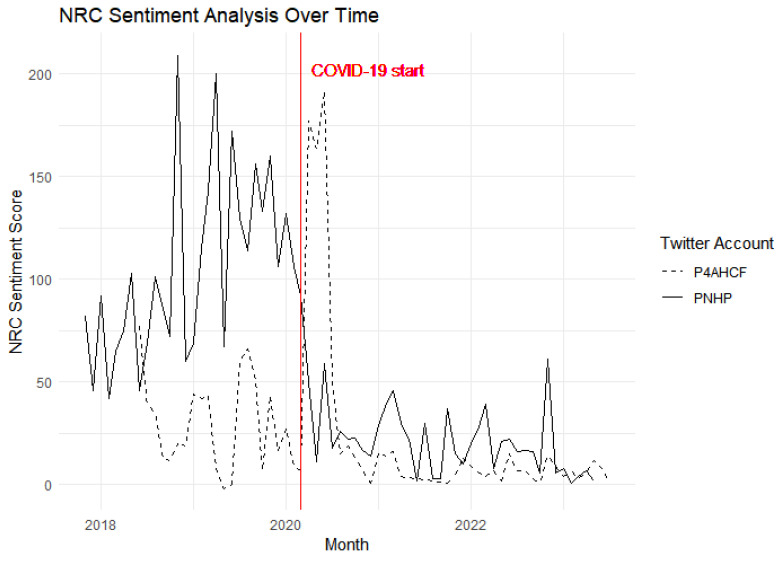
NRC Sentiment Scores across time.

**Figure A4:**
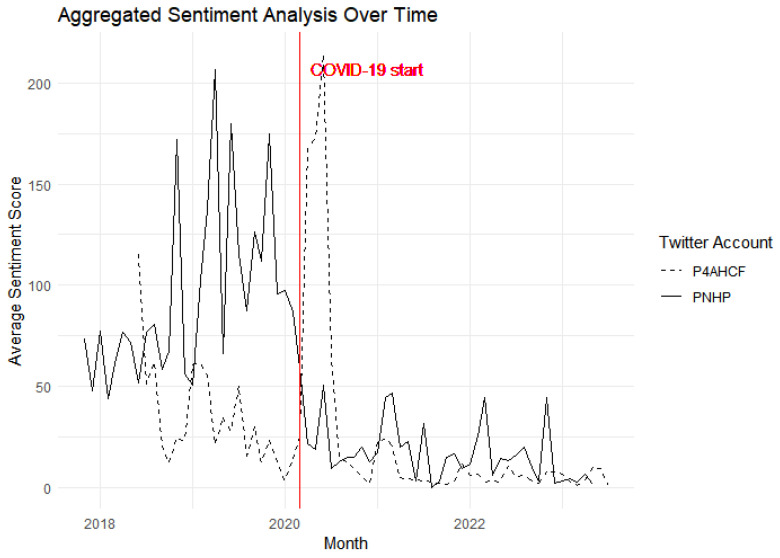
Aggregated Sentiment Scores across time.

## Data Availability

All data and codes used for this study are publicly available and can be downloaded from the Harvard Dataverse using the link https://doi.org/10.7910/DVN/OWVD70.
